# Optimized RNA Extraction and Northern Hybridization in Streptomycetes

**DOI:** 10.1007/s12575-010-9027-7

**Published:** 2010-03-17

**Authors:** Marcello Tagliavia, Anna Taravella, Sandra Marineo, Anna Maria Puglia, Mario La Farina

**Affiliations:** 1 Dipartimento di Biologia Cellulare e dello Sviluppo, Università di Palermo, Viale delle Scienze (Parco d'Orleans), Edificio 16, 90128, Palermo, Italy

**Keywords:** streptomycetes, total RNA purification, RNA processing, RNA degradation, RNA glyoxylation, alkaline blotting, northern hybridization

## Abstract

Northern blot hybridization is a useful tool for analyzing transcript patterns. To get a picture of what really occurs in vivo, it is necessary to use a protocol allowing full protection of the RNA integrity and recovery and unbiased transfer of the entire transcripts population. Many protocols suffer from severe limitations including only partial protection of the RNA integrity and/or loss of small sized molecules. Moreover, some of them do not allow an efficient and even transfer in the entire sizes range. These difficulties become more prominent in streptomycetes, where an initial quick lysis step is difficult to obtain. We present here an optimized northern hybridization protocol to purify, fractionate, blot, and hybridize *Streptomyces* RNA. It is based on grinding by a high-performance laboratory ball mill, followed by prompt lysis with acid phenol-guanidinium, alkaline transfer, and hybridization to riboprobes. Use of this protocol resulted in sharp and intense hybridization signals relative to long mRNAs previously difficult to detect.

## 1 Introduction

Northern hybridization, although less sensitive than other methods for RNA analysis, is the only technique providing information about the concentration of specific transcripts among a complex, overlapping RNA population. This information is required in the study of important events of gene expression regulation, such as RNA transcription termination, processing, and degradation. The quality of a northern hybridization protocol depends on three main points: protection of RNA integrity, unbiased recovery of the entire transcripts population, and its efficient and even transfer to the filter.

Streptomycetes are largely studied antibiotic-producing soil bacteria, which undergo a complex life cycle characterized by the differentiation of a vegetative and an aerial, spore-producing mycelium. Lysis of these and of other gram-positive organisms is difficult to achieve. Most *Streptomyces* RNA purification protocols include an initial incubation step during which some RNA degradation pathways active in vivo may keep on going in vitro. This often results in RNA degradation. To overcome these artifacts, an efficient lysis must be promptly achieved and quickly followed by addition of a fully denaturing agent which protects RNA integrity.

A second point that must be considered is the loss of low M.W. transcripts occurring when the RNA is purified by column chromatography.

Finally, blotting in the presence of a neutral buffer results in a non-efficient transfer of high M.W. transcripts to the filter.

Thus, we set up an optimized protocol for studying mRNA processing and decay in streptomycetes which could overcome these limits. This procedure allowed to gain information on the expression of *Streptomyces coelicolor dnaK* operon, indicating that early published data (see Figure 2 in [1], probably reflected a selective loss of specific transcripts leading to a misinterpretation of what really occurs in vivo.

**Figure 2:**
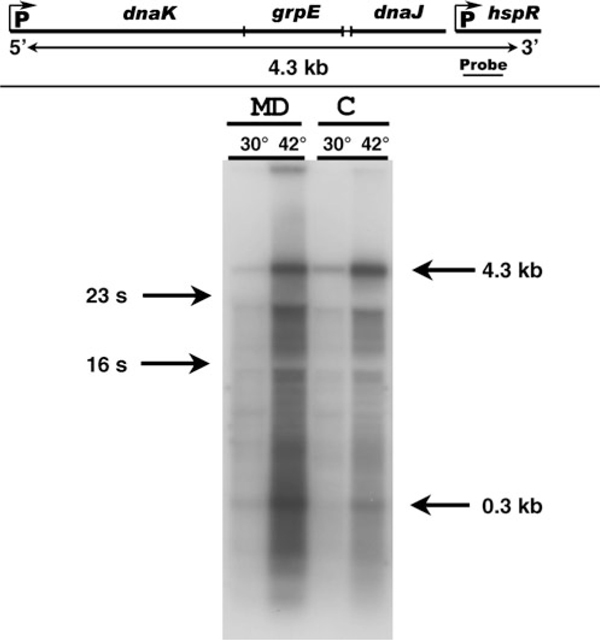
**Hybridization to a probe complementary to the distal region of *dnaK* operon**. The nylon filter was hybridized to a riboprobe as described in "Materials and Equipment" and "Methods" sections. The *dnaK* operon map shown in the *upper part* of the figure was that of [9] modified according to [6].

## 2 Materials and Equipment

R5 Agar: 103 g/l sucrose, 0.25 g/l K_2_SO_4_, 10.1 g/l MgCl_2_.6H2O, 10 g/l glucose, 0.1 g/l casamino acids, 5 g/l yeast extract, 5.72 g/l TES, H_2_O to 900 ml. After autoclaving, 0.5 mg/l K_2_PO_4_, 15 mg/l CaCl_2_.2H_2_O, 3 g/l L-prolin, 2.8 g/l NaOH, 8 ml/l trace elements solution and H_2_O to 1 l were added. Trace elements solution contains: 40 mg/l ZnCl_2_, 200 mg/l FeCl_3_.6H_2_O, 10 mg/l CuCl_2_.2H_2_O, 10 mg/l MnCl_2_.4H_2_O, 10 mg/l Na_2_B_4_O_7_.10H_2_O, 10 mg/l (NH_4_)Mo_7_O_24_.4H_2_O.

Cellophane (Bio-Rad)

**Table:** 

SolD	4 M guanidinium isothiocyanate, 0.025 M sodium citrate, 5 g/l sarkosyl. Immediately before use, 2-mercaptoethanol (Merck) to a final concentration of 0.1 M was added.

Phenol (USB) saturated with H_2_O.

Chloroform

**Table:** 

BTPE 10×	60 g/l BisTris (USB), 30 g/l PIPES, 20 ml/l 0.5 M EDTA (USB) pH 8, H_2_O to 1 l.
SSPE 20×	175.3 g/l NaCl, 27.6 g/l NaH_2_PO_4_, 9.4 g/l Na_2_ EDTA, pH 7.4
Glyoxal mixture	600 ml/l DMSO, 1.2 M glyoxal, 48 ml/l glycerol, 0.2 mg/ml ethidium bromide (USB).

The transfer apparatus consist in filter paper, Whatman 3MM filter paper, and nylon membrane ZetaProbe (BioRad).

RNeasy Kit (Qiagen)

Dnase I (Invitrogen)

Chemicals were from Sigma, where not otherwise specified.

## 3 Methods

### Bacterial cultures

Dense spore suspension (100 μl; ca. 10^8^ spores ml^-1^) of *S. coelicolor* M145 was plated directly onto sterile cellophane covered R5 plates [2] and incubated at 30°C. After 20-h growth, half of the culture plates were heat shocked at 42°C and half were left at 30°C. Fifteen minutes later the mycelium was promptly scraped from the cellophane, immediately frozen in liquid N_2_ and ground in Mikrodismembrator II (Sartorius) at 2,000 rpm for 45 s (alternatively, mycelium can be stored at -80°C).

### RNA extraction

The mycelium powder was treated according to [3] with some modifications. It was suspended in prewarmed (about 50°C) SolD (approximately 1.5 ml/100 mg mycelium), the solution was then transferred into a Corex glass tube and 0.01 vol of sodium acetate 2 M pH 4 and 1 vol of acid phenol were added, mixing thoroughly after each addition. After few minutes, 0.2 vol of chloroform were added, and after vortexing and a 15-min incubation, the sample was centrifugated at 15,000 × g for 30 min. To the aqueous phase, 1 vol of isopropanol was added and RNA was recovered by centrifugation after at least 1 h at -20°C, suspended in 0.33 vol of SolD and precipitated with 1 vol of isopropanol at -20°C for 45 min. After centrifugation, RNA was suspended in BTPE 0.5×, treated with DNase I (Invitrogen), phenol/chloroform extracted, precipitaded with 0.33 vol of ammonium acetate 10 M and 2.5 vol of ethanol at -20°C and after centrifugation, suspended in BTPE 0.5×.

The extraction using RNeasy Kit (Qiagen) was carried out accordingly to the manufacturer's instructions.

### Northern

RNA was glyoxylated using 10 μl glyoxal mixture/2 μl RNA [4]. The sample was incubated at 55°C for 1 h, chilled on ice, and loaded onto 1.8% agarose gel in BTPE 1×. The fraction action was carried out at 5 V/cm.

After electrophoresis the gel was soaked in 30 mM NaOH for 5 min with gentle agitation, then put onto downward transfer apparatus, using 20 mM NaOH as transfer solution. After 2 h, the gel was removed, the filter air dried for 1 min, neutralized in BTPE/50 mM Tris-acetate pH 7, air dried, UV_254nm_ fixed for 15 s, and hybridized to a ^32^P-labeled riboprobe (spec. act. 7 × 10^8^ cpm/μg) in PerfectHyb Plus Hybridization Buffer (Sigma), at 73°C overnight. After hybridization, the membrane was washed twice in 0.4× SSPE at 73°C and twice in 0.1× SSPE at 67°C.

**Table:** 

Northern RNA protocol
1. Grow bacteria on cellophane covered plates.
2. Harvest mycelium by scraping and promptly freeze it by immersion in liquid N_2_.
3. Grind the frozen sample in Mikrodismembrator for 45 s at 2,000 rpm (alternatively, it can be stored at -80°C).
4. Extract RNA according to [3]. Prewarm SolD and use larger volumes (1.5 ml/100 mg mycelium).
5. Further purify RNA by ethanol precipitation in the presence of 2.5 M ammonium acetate.
6. Glyoxylate and fractionate the RNA according to [5].
7. Soak the gel in 20 mM NaOH for 5 min in agitation.
8. Transfer the RNA to a nylon membrane by alkaline downward blotting according to [6].
9. Hybridize the filter to an antisense riboprobe.

## 4 Discussion of Key Steps in the Protocol

The first crucial step in the present protocol is the grinding of the frozen mycelium. Grinding of the mycelium, previously frozen in liquid N_2_, by means of Mikrodismembrator II (MD) reduces it, maintained in a frozen state, to a fine and homogeneous powder in a very short time (45 s) thus preventing all degradative processes. MD is a small apparatus where a small steel ball is moved in a bilateral direction inside a small grinding chamber, successfully used for nucleic acids extraction from various biological samples ranging from tumoral tissues [7] to *Bacillus subtilis* cells [8]. To maintain the mycelium in a frozen state during grinding, the plastic chamber must be prechilled with liquid N_2_. This precaution allows fast, efficient, and highly reproducible grinding of *Streptomyces* mycelium. The RNA is then purified according to [3]. The use of prewarmed SolD to solubilize the mycelium powder prevents the freezing of this buffer and/or the precipitation of some components following the contact with the frozen sample thus allowing a prompt denaturation of cellular RNases. Our purification protocol does not make use of columns thus preventing loss of low M.W. RNA, and the A_260/280_ of purified RNA appears to be equivalent to that obtained by column procedures. Moreover, the use of an alkaline medium for transfer of the RNA to a filter [5] improves, by partial RNA hydrolysis, the transfer of high M.W. transcripts. We optimized this step by a preliminary soaking of the gel in NaOH, which allows the gel pH to quickly turn from 6.5 to at least 9.0. Moreover, the use of a NaOH concentration higher than that suggested by [5] improves glyoxal removal and hydrolysis of long RNA molecules resulting in their efficient transfer to the filter and stronger hybridization signals.

**Figure 1:**
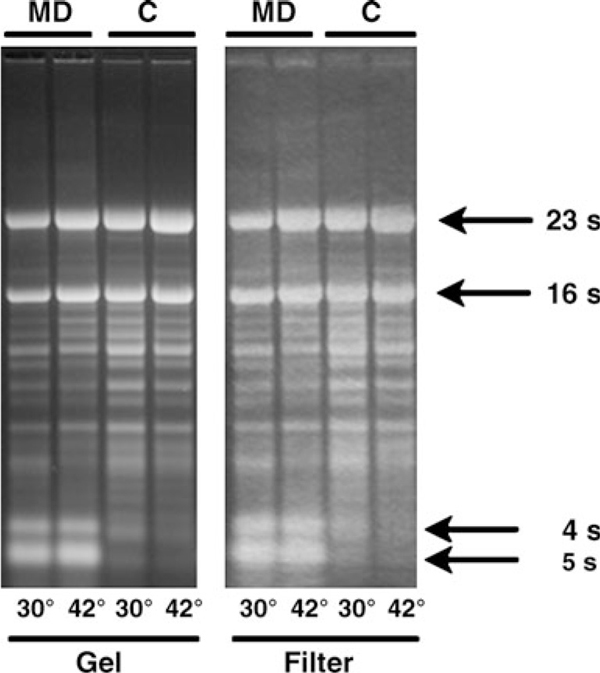
***Streptomyces* RNA in agarose gel and onto nylon membrane**. Glyoxylated RNA (10 μg; purified by means of the Mikrodismembrator (*MD*) or the columns (*C*) protocol were fractionated on an agarose gel and transferred to a nylon membrane as described in "Materials and Equipment" and "Methods" sections. The *two panels* show, respectively, the bands visualized in the gel after fractionation and on the nylon filter after the RNA transfer. Pictures were taken under UV_260_ light.

We applied our protocol to the analysis of RNA complementary to the *dnaK* operon of *S. coelicolor*. Figure 1 shows the pattern of ethidium bromide stained bands seen on the gel and on the filter after the RNA transfer. It clearly appears that the bands corresponding to the 4S and 5S RNA species are almost completely absent in the samples purified by the column procedure (C). However, the intensity of the bands corresponding to larger species is equivalent in the two sets of samples. Figure 2 shows the bands seen after hybridization of the filter to an antisense riboprobe complementary to the 3' distal region of *dnaK* operon. This probe hybridizes with transcripts ranging from 4.3 Kb (corresponding to the entire operon [1] to about 300 nt [6]. Their expression level is much more enhanced when the temperature of growth is raised from 30°C to 42°C (heat shock treatment [1,6]. Comparing our results with those already published on the RNA population complementary to the *dnaK* operon during the heat shock response [1] it clearly appears that the use of our protocol results not only in a highly enhanced intensity of bands associated with the largest transcripts but also in an inversion of the relative intensity of large and small transcripts. Figure 2 shows that the relative intensity of the 4.3 Kb transcript is almost equivalent in samples purified according to either of the two protocols. However, it clearly appears that the hybridization signal of low M.W. transcripts present in the RNA purified with our procedure is much more intense than that seen in the homologous C sample. The latter, although purified by a protocol defined for total RNA extraction (claimed cut-off at 200 nt), results in loss of medium-low M.W. transcripts.

All this stresses the quality of our protocol for studying dynamic events of RNA metabolism where the relative concentration of high and low M.W. transcripts must be compared.
